# Facility readiness and counseling during antenatal care and the relationship with early breastfeeding in Haiti and Malawi

**DOI:** 10.1186/s12884-020-02919-7

**Published:** 2020-05-29

**Authors:** Lindsay Mallick, Rukundo K. Benedict, Wenjuan Wang

**Affiliations:** 1 The DHS Program, Avenir Health, ICF, 530 Gaither Road, Suite 500, Rockville, MD 20850 USA; 2 The DHS Program, Rockville, MD USA

**Keywords:** Early initiation of breastfeeding, Breastfeeding counseling, Antenatal care, Service readiness, Service Rrovision Assessment (SPA), Demographic and Health Survey (DHS)

## Abstract

**Background:**

Early initiation of breastfeeding (within an hour of birth) has benefits for newborn health and survival. Optimal breastfeeding supports growth, health, and development. Health facilities provide essential pregnancy, maternal, and newborn care and offer support for early breastfeeding. We examined the relationship between the breastfeeding-related health service environment during antenatal care (ANC) and early initiation of breastfeeding.

**Methods:**

Using data from recent Service Provision Assessment (SPA) surveys in Haiti and Malawi, we defined three indicators of the health service environment: availability of facilities with ANC services reporting routine breastfeeding counseling; provider training on breastfeeding; and breastfeeding counseling during ANC. We linked SPA data geographically to Demographic and Health Surveys (DHS) data from Haiti and Malawi. Multilevel, multivariable logistic regressions examined associations between the health service environment and early initiation of breastfeeding, controlling for women’s background characteristics, with separate analyses for urban and rural residence.

**Results:**

Over 95% of facilities in Haiti and Malawi reported routinely providing breastfeeding counseling during ANC. Only 40% of both urban and rural providers in Malawi and 29 and 26% of providers at urban and rural facilities in Haiti (respectively) received recent training in counseling on breastfeeding. Further, only 4–10% of clients received counseling. Breastfeeding counseling was generally more common among clients who attended ANC with a provider who had received recent training. After linking SPA and DHS data, our analysis showed that having more providers recently trained on breastfeeding was significantly associated with increased odds of early breastfeeding among women in urban areas of Haiti and Malawi. Additionally, women in urban areas of Malawi lived near facilities with more counseling during ANC were more likely to begin breastfeeding within an hour of birth compared with women in areas with less counseling.

**Conclusions:**

Our study identified gaps in the health system’s capacity to implement the recommended global guidelines in support of optimal breastfeeding practices. While breastfeeding counseling during ANC can promote early breastfeeding, counseling was not common. The study provides evidence that provider training could help improve counseling and support for early initiation of breastfeeding.

## Background

Breastfeeding supports children’s health, growth, and development and could prevent over 800,000 deaths of children under age 5 annually [1, 2]. The World Health Organization (WHO) recommends early initiation of breastfeeding (EIBF), defined as initiating breastfeeding within the first hour of birth [3]. EIBF protects infants from infection and death and promotes exclusive breastfeeding [4, 5]. Despite the benefits of EIBF, global progress has been slow. In 2018, only an estimated 42% of children began breastfeeding within an hour of birth [3, 6, 7].

Breastfeeding practice is influenced by individual, household, community, socioeconomic, health system, and policy factors, including birth size, maternal education, wealth, residence, antenatal care (ANC) attendance, skilled birth attendance, and mode of delivery [1, 8–14], although these associations may vary by urban and rural residence [8, 15]. There are a number of interventions that are known to target many of these factors in order to improve EIBF [1, 16]. Among these, health systems interventions are the most effective, yet they have a stronger effect in low- and middle-income countries than high income countries, as well as in rural areas than urban, according to meta-analyses by Sinha et al. [16].

One such intervention, the Baby-Friendly Hospital Initiative (BFHI), works to protect, promote, and support optimal breastfeeding practices in health facilities that provide maternal and newborn care services through implementation of the Ten Steps to Successful Breastfeeding, including ANC services [3]. BFHI and other structured facility programs have been shown to improve breastfeeding practices compared with standard care [17]. In a systematic review that included low-, middle- and high-income countries, a dose-response relationship between each additional BFHI step and improved levels of EIBF was identified [18]. Despite the evidence, BFHI adoption, adherence, and implementation are suboptimal in many countries across most regions [19].

Although the health service environment is an important factor for EIBF, there are few large-scale studies, especially in low- and middle-income countries, that have examined service environment factors related to quality of care. Khan et al. [11] examined characteristics of country-level health systems, including government health expenditure and health-worker-to-population ratios, identifying bivariate associations with EIBF in a pooled analysis of 15 countries. Another study by Takahashi et al. [20] found that facility characteristics such as location, capacity, ownership, and number of beds were not associated with early breastfeeding, whereas having guidelines for postnatal or neonatal care was associated. The authors postulate that guidelines may be a proxy for quality of care or service delivery, and that quality of care may be a stronger determinant of EIBF than other facility characteristics which are not reflective of quality of care [20]. However, there is a dearth of population-based studies examining the relationship between service delivery of breastfeeding counseling during ANC and early initiation of breastfeeding.

The aim of this study is to first describe the breastfeeding-related service environment including facility readiness, provider training, and the delivery of services during ANC in Haiti and Malawi. Second, this study links nationally representative health facility data from Service Provision Assessment (SPA) surveys with household-based Demographic and Health Surveys (DHS) data to assess the relationship between the breastfeeding-related service environment and EIBF. SPA surveys are cross-sectional, nationally-representative surveys conducted at the request of countries on a periodic basis (usually every 5 years) to assess health facilities in a given country. SPA surveys can be used to provide insight about the three components of quality of care—*structure, process, and outcome—*as defined by Avedis Donabedian [21, 22]. The *structure* component describes the availability and readiness (capacity) of facilities to provide the service, including trained providers. For select services such as ANC, the SPA includes an observation of visits as well as client exit interviews. This information collected during the visit can be used to describe Donabedian’s process component of the quality of care, including counseling provided during the service. SPA surveys also provide limited information about the outcome component, including client satisfaction with services.

We chose to analyze data from Haiti and Malawi given the unique data available in each county. Both countries have conducted recent, closely timed DHS and SPA surveys. Further, the SPA surveys conducted in Haiti and Malawi were censuses—that is, they included every formal health sector facility rather than just a sample. This census design enables geographic linkage of the health facility data to population data in DHS enumeration areas (clusters), described below and in Burgert and Prosnitz [23]. Although we make some comparisons throughout the text in order to summarize results, these countries have both different norms around breastfeeding and different health systems landscapes. Thus, comparisons should be interpreted with an understanding of the different contexts, described below.

## Country context

### Haiti

Haiti is a low-income country with a quarter of Haitians living below the international poverty line of $1.90 a day [24]. The country relies on donor funding for almost half their health spending [25]. The health sector is heavily impacted by a shortage of trained health providers [26] and weak infrastructure. The health sector is organized into three hierarchical levels. At the primary level, dispensaries provide basic outpatient care such as preventative and curative services. At the secondary level, health centers provide basic services, community programs, and in and outpatient procedures. Referral hospitals are the tertiary level and provide advanced medical procedures in addition to lower level care [27].

In Haiti, less than 1% of facilities have ever been BFHI certified by the national government, and information on recertification efforts is sparse [28, 29]. Rates of EIBF in the country have remained relatively unchanged, at 47%, between 2000 and 2017 [30]. In the most recent DHS survey, there were marginal differences in EIBF by place of delivery, skilled assistance at birth, but some variation by wealth and education, where EIBF was more common among lower wealth quintiles and lower maternal education compared with higher wealth and education [31]. The 2013–18 Haiti National Nutrition Strategy Plan outlined six strategic areas to address malnutrition and coordination of multisectoral approaches, including breastfeeding support; however, regional monitoring and implementation of the BFHI has been slow and fragmented [31, 32].

### Malawi

Similar to Haiti, Malawi is a low-income country with almost three quarters of its population living below the international poverty line. Over half Malawi’s health expenditure is from donors and the country also experiences shortages of trained health providers and poor infrastructure [25]. The organizational structure of Malawi’s heath sector is similar to Haiti and comprises lower level facilities such as dispensaries and health posts at the primary level, district hospitals at the secondary level, and central hospitals at the tertiary level [33].

The rates of EIBF in Malawi are higher than the global average, and have increased from 70% in 2000 to 94% in 2010 but declined to 76% by 2015–16 [30]. The most recent DHS survey showed there were minor differences in EIBF by place of delivery, where 77% of children born in a facility breastfed in the first hour versus 68% among those born at home. Similar to Haiti, babies born to mothers of lower wealth or lower educational attainment were more often breastfed in the first hour compared with highest socio-economic characteristics. The Malawi government was an early adopter of the BFHI in 1993 [34]. Through the 2007–12 Malawi National Nutrition Policy and Strategic Plan and subsequent 2018–2022 National Multi-Sector Nutrition Policy, the government outlined its multisectoral response to address malnutrition, including promotion of optimal breastfeeding at the facility, community, and household levels [35, 36]. By 2008 almost 5 % of facilities were ever certified, but a 2015 evaluation of facilities revealed there were no longer any BFHI certified facilities in the country [34, 37]. Although funding constraints hampered the implementation of the BFHI in the mid-2000’s, recently the government has re-focused on strengthening the BFHI including certification of facilities [34].

## Data and methods

### Data

This paper uses publicly available data (https://www.dhsprogram.com/) from the 2013 Haiti SPA [27] and the 2013–14 Malawi SPA [33], together with data from DHS surveys conducted within 2–3 years of the SPA, in 2016–17 in Haiti and 2015–16 in Malawi [38, 39]. The ICF Review Board and the Ethics Review Committee of the countries included in this study reviewed and approved the respective surveys. More information about the survey process, such as the complex survey design and response rates, can be found in corresponding survey final reports [29–32]. We link data from the SPA with DHS data within the respective countries in order to explore the relationship between the local health service environment and women’s practice of EIBF. SPA surveys provide nationally representative health facility data on service provision for key health services. Facility data from formal sector public, private (for-profit, not-for-profit, nongovernmental, faith-based), and mixed private-public facilities are included, while data from informal outlets such as pharmacies and mobile clinics usually are excluded. The SPA collects data on the availability and readiness to provide services, e.g., basic infrastructure, equipment, medicines, guidelines, and human resources at the facility as well as provider experience, qualifications, training, and routine duties, and perceptions of the service environment. SPA surveys also record the content and quality of selected healthcare visit for services including ANC—up to 15 consultations per service per facility—and includes exit interviews, conducted with clients whose visit was observed to learn their perceptions on the visit.

In this study, we used data from facilities that provide ANC services, including 310 urban and 522 rural facilities in Haiti and 116 urban and 516 rural facilities in Malawi. We examined data on providers at these ANC facilities who provide care that could encompass counseling on breastfeeding practices (antenatal, postnatal, newborn, or child health care). We also used client data from observations of women attending ANC visits, including the records of the observation and the exit interview. Table 1 presents the sample of facilities, providers, and clients in Haiti and Malawi included in the analysis.

**Table 1:** Samples included in the analysis

	Haiti			Malawi		
	Urban^a^ N (%)	Rural N (%)	Total N	Urban N (%)	Rural N (%)	Total N
**SPA survey**						
Year of survey	2013			2013–14		
Number of facilities with ANC	310 (37)	522 (63)	832	116 (18)	516 (82)	632
Number of health providers	707 (48)	758 (52)	1465	326 (29)	783 (71)	1109
Number of ANC clients observed	945 (58)	675 (42)	1620	587 (28)	1481 (72)	2068
**DHS survey**						
Year of survey	2016–17			2015–16		
Number of women^b^	357 (22)	1278 (78)	1634	772 (13)	5296 (87)	6067

^a^Urban includes all urban facilities. However, analysis of women excludes urban women living in Port-au-Prince or rural women within 5 km of Port-au-Prince

^b^Women analyzed included women with a live birth in the last 2 years, had at least one antenatal care (ANC) visit during their last pregnancy resulting in a live birth in last 2 years, delivered vaginally, and whose newborn survived the first day or longer

Table 1 also shows the number of women studied from the DHS. The DHS Program conducts surveys among households in low- and middle-income countries approximately every 5 years. Data are representative nationally as well as subnationally by urban and rural residence and by region. Surveys are implemented with a two-stage cluster sampling design. We conducted the analysis among women age 15–49 who had a live birth in the 24 months preceding the survey. For women with more than one birth in the time period, we examined only the most recent pregnancy and live birth. We excluded women who delivered via cesarean section (C-section) and women whose babies who died on the first day of life, as the mother-baby dyad faced complications that may have precluded the ability to breastfeed [20, 40], although this did not eliminate the chance entirely. As the SPA data describe the healthcare provided at facilities offering ANC, we examined data only for mothers who attended ANC at least once during the index pregnancy, to ensure that the population studied was applicable to our linked service environment [41]. In sum, the analysis comprised 357 urban and 1278 rural women in Haiti and 772 urban and 5296 rural women in Malawi.

## Methods

### Health service environment indicators

To assess the health service environment, we first identified three indicators of quality of care or readiness to provide care relative to breastfeeding among the three different populations or units assessed by the SPA surveys: facilities, providers, and clients. Among facilities that provide ANC, readiness included two variables. First, we examined the proportion that reported routine counseling on breastfeeding as part of ANC services. For the provider indicator, we constructed a variable based on information collected in the SPA health worker questionnaire to assess whether the provider had received any recent training related to breastfeeding. Specifically, we examined the proportion of health workers who provide ANC services and who received training within the past 24 months on breastfeeding, infant and young child feeding (IYCF), or early and exclusive breastfeeding.

For the client variable, we combined data from both the observation of ANC and the client exit interview data. We defined this a client-level variable as the proportion of clients who were observed receiving counseling on breastfeeding (early initiation, prolonged breastfeeding, or exclusive breastfeeding) and who also reported in the exit interview having received counseling on breastfeeding during the observed visit. Research shows not all client reports (as assessed in exit interviews) accurately recall and portray counseling provided [42] and the client exit interview is subject to courtesy bias, where clients may over-report receipt of counseling [43, 44]; prior research therefore recommends constructing a variable that combines records from the interviewer’s observation and client report [43]. Thus, to reduce the effects of courtesy bias and recall bias, we positively coded this variable based on concordant responses—if both the recorded observation of counseling and the client’s account of counseling agreed. We presented each of the three indicators by urban and rural location of facilities in each country. We further presented the percentage of clients counseled according to recent provider training—for each type of training individually as well as the combined indicator for any training related to breastfeeding—and tested the differences in counseling using chi square tests of independence.

### Linking SPA and DHS surveys

In order to test the association between the health service environment factors and early initiation of breastfeeding, we linked the facility-level data in the SPA surveys to clusters of women interviewed in DHS surveys in Haiti and Malawi. To enable linking, we summarized the provider- and client-level indicators within each facility. We merged provider information with facility data, summarizing data about all providers at a given facility to create an indicator for number of providers with recent training related to breastfeeding within each facility. For clients, we again merged client data with facility data. We tabulated the overall proportion of clients counseled at each facility among all facilities and then dichotomized facilities as either below the overall average or average to above average proportions of clients counseled.

After creating facility-level variables for provider and client data, we then summarized all of the facilities within geographic ‘zones’ around DHS clusters in order to facilitate linking. We created these zones of facilities within a 10 km radius for rural clusters and a 5 km radius for urban clusters, using GPS coordinates of facilities and clusters [23]. Creating zones of facilities using these distances are necessary as The DHS Program displaces GPS coordinates of sampled clusters. This linking process has been described in previous studies [45].

We used a slightly different summary calculation for each service environment indicator within each zone. For the service environment indicator for access to health facilities offering ANC that report conducting breastfeeding counseling, we calculated the number of facilities within each zone. For the indicator of access to trained providers, we calculated the average number of recently trained providers at ANC facilities within the zone. For counseling on breastfeeding, we averaged the proportions of clients reporting and observed receiving counseling among facilities within each zone.

We categorized clusters as having no data on facilities within the zone, or as having a low, medium, or high level of service environment for each indicator using tercile cut points for each urban and rural cluster classification. Although using a continuous measure of the service environment would have been preferable, we categorized clusters in order to maintain the sample of clusters for which there were either no ANC facilities that report routine counseling services, no ANC providers interviewed at the ANC facilities, or no facilities with observations of ANC client visits. Thus, we could not leave the score as a continuous measure otherwise these clusters would have been either scored as zero or excluded altogether, which could consequently bias the results or reduce our power. For instance, some women with no health facility near their home might not be able to seek care because they lacked access, but others might seek care from a facility farther away. Further, although facilities without observations of any client visits tend to have less structural readiness [46], it is uncertain, had there been any clients observed in these facilities, whether providers would have counseled them on breastfeeding; service readiness is not necessarily associated with the quality of care provided [47]. Highly skewed quality indicators, when linked to clusters, did not allow for tercile categorization. In these cases, the medium category was dropped, leaving categories of no data, and low or high only.

Given the population density of Port-au-Prince (27,395/km^2^ within 36 km2 [48]), and the high concentration of facilities in the capital [49], where nearly one fifth of all facilities (174 of 905 facilities) are located [27], urban women in this city were excluded from the analysis; the 5 km radius might not be precise enough to define the service environment in this setting, where women have greater access to a larger number of facilities. We also excluded clusters of rural women within 5 km of Port-au-Prince, given their proximity to many urban facilities. Thus, 64 clusters of 398 women were excluded from the analysis in Haiti. We therefore describe the urban population in Haiti as “other urban”.

### Analysis

Our outcome of interest was early initiation of breastfeeding, which was based on responses to a question asked to mothers who ever breastfed, “*How long after birth did you first put (NAME) to the breast?*” If a woman responded immediately or within the first hour, we defined this as early initiation of breastfeeding.

We analyzed the bivariate and multivariable relationships of early initiation of breastfeeding and the health service environment. We first examined the coverage of EIBF among women by the service environment in their nearby health facility offering ANC—that is, we measured the proportion of women who began breastfeeding within an hour of birth by whether they resided in a cluster with a low, medium, high, or “no data” service environment, based on the three service environment variables: number of proximate facilities (within the respective radius) offering ANC that reported routine counseling on breastfeeding, average number of trained providers within the radius around the cluster, and average proportion of clients counseled. We then conducted multilevel, multivariable logistic regression, by urban (or other urban) and rural residence. Multilevel logistic regression models account for the fact that women who live near on another are not necessarily independent of one another; the models simultaneously test for the effects of both group-level and individual-level effects on individual-level outcomes. In this study, the service environment variables represent group-level factors, while sociodemographic and care-seeking variables are individual-level factors.

Our regression analysis controlled for sociodemographic and care-seeking characteristics of the mother and baby. Maternal characteristics comprised region, wealth quintile regrouped into three categories (lowest and second; middle; fourth and highest), education (no school or primary; secondary or higher), employment (not employed; employed), religion (Christian; other), media exposure (exposed to TV, radio, or newspaper less than once a week; exposed to TV, radio, or newspaper at least once a week), marital status (not married; currently married), and parity (primiparous; multiparous). Characteristics of the child described sex at birth (male; female) and perceived or recorded size at birth (normal; smaller than average; larger than average), where mother’s perception of size was used in the absence of a reported or documented birth weight. Care-seeking variables included number of ANC visits (one to four; four or more), facility delivery by a skilled birth attendant (yes; no), and postnatal check, a proxy for postnatal care (PNC), for either the mother or baby within the first hour of birth (yes; no). A postnatal check includes whether or not a provider, community health worker, or traditional birth attendant checked on the health of either the mother or the baby within the first hour of birth. Although there exists a question specific on postnatal counseling and observation of breastfeeding in the DHS surveys, the time period noted refers to 2 days after delivery. As our outcome refers to the first hour after delivery, we could not include this as a variable without the risk of the predictor variable proceeding the outcome.

## Results

The characteristics of the facilities varied by country and urban and rural location in terms of the type of facility (hospital; health center; and dispensary, clinic or other), managing authority (public, private nonprofit, and private for profit or other), and department or region of the country (Additional file Table 1). In Haiti, hospitals are university, regional, community, and other hospitals; and health centers include those with and without beds. In Malawi, hospitals are central, district, community, and other hospitals; health centers include maternity health centers; and other refers to health posts. In Haiti, health centers constituted the majority of facilities in urban areas (65%) while in Malawi, facilities comprised mainly health centers in rural areas (80%) and were the least common in urban areas (24%). Facilities were evenly distributed by managing authority in urban areas of both countries, although in rural areas of both countries, government facilities were the most prevalent (44% in Haiti and 67% in Malawi).

### Readiness to offer counseling on breastfeeding during ANC and provision of counseling

**Fig. 1:**
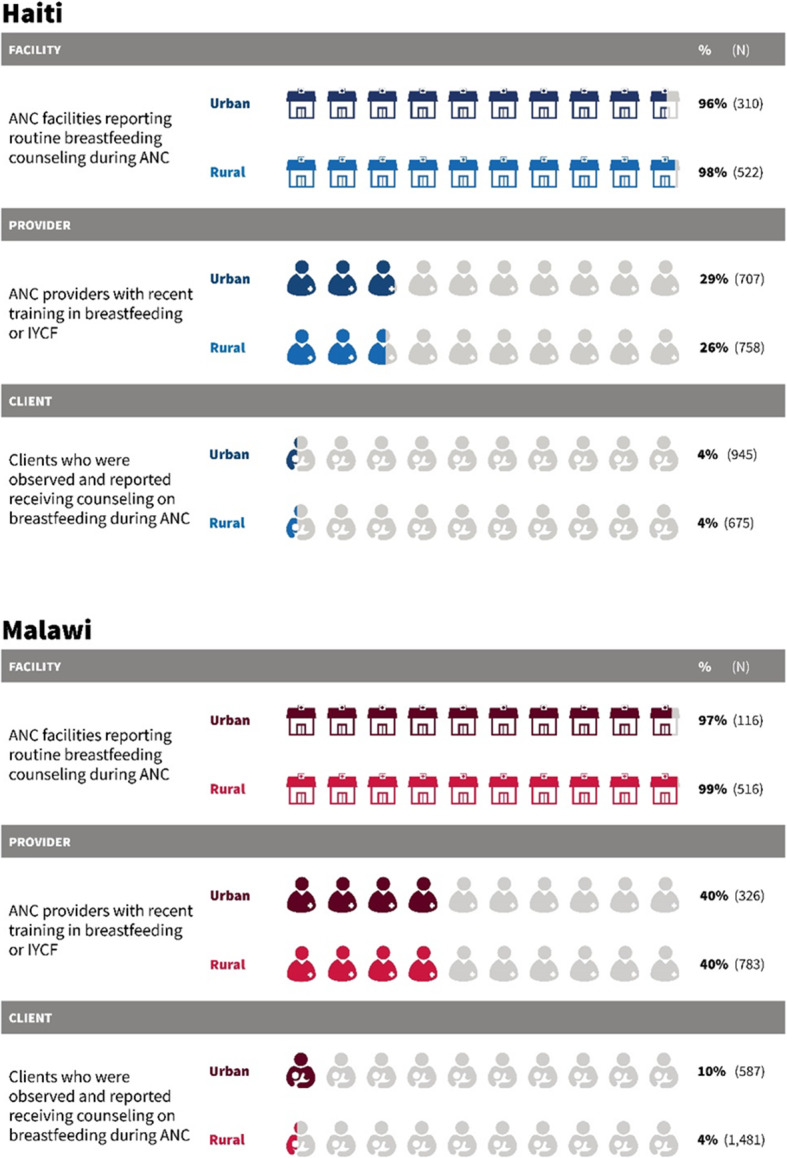
Readiness to provide breastfeeding counseling and provision of counseling among facilities, providers, and clients, Haiti and Malawi

Figure 1 shows the percentage of facilities with readiness to offer counseling on breastfeeding, the level of training on breastfeeding among providers, and counseling about breastfeeding provided during ANC, by urban and rural location in Haiti and Malawi. In Haiti, among facilities that provide ANC, 96% of urban facilities and 98% of rural facilities reported that they routinely provide counseling about breastfeeding as part of ANC services. Twenty-nine percent of ANC providers at urban facilities and 26% at rural facilities reported having attended recent training related to breastfeeding or IYCF. While almost all facilities in Haiti reported that counseling on breastfeeding was a routine practice during ANC, only 4% of both urban and rural clients in fact were observed to have received counseling during ANC and reported receiving it. As counseling on breastfeeding increases in relevance to ANC visits attended later in pregnancy [50], we calculated the proportion of women counseled among women attending ANC in their third trimester (*N* = 557) and found that the proportion was still only 5% (results not shown). Even among women 36–41 weeks pregnant, only 5% were counseled and reported having received the counseling (*N* = 140).

Malawi shows similar findings for routine breastfeeding counseling, training on breastfeeding, and counseling on breastfeeding during ANC. Nearly all facilities reported that breastfeeding counseling is part of their routine care during ANC, at 97 and 99% in urban and rural facilities respectively. In rural and urban areas alike, 4 out of every 10 ANC providers reported recent training related to breastfeeding or IYCF. Also similar to Haiti, few clients were counseled about breastfeeding during ANC—10% of clients attending urban facilities and 4% of clients attending rural facilities. In Malawi as well, we examined the proportion of women counseled in the third trimester (*N* = 1208) and at 36–41 weeks pregnant (*N* = 210) and, as in Haiti, found that only 5 and 4% of clients, respectively, were counseled (results not shown).

**Fig. 2:**
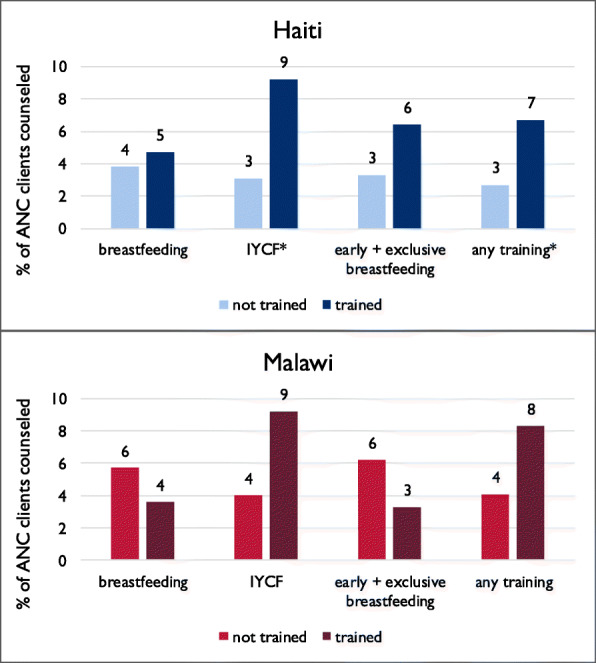
Percentage of antenatal clients counseled by provider training according to Service Provision Assessment observations, Haiti and Malawi

Figure 2 shows the percentage of ANC clients counseled on breastfeeding by whether their provider had received recent training related to breastfeeding, including general breastfeeding, IYCF, early and exclusive breastfeeding, or the composite indicator of any one of those trainings. In Haiti, for each type of training as well as the composite indicator, levels of counseling were higher among clients whose provider had recent training, with significant differences for training related to IYCF and for any relevant training (*p*-value < 0.01). The proportion of clients counseled was three times higher among clients who saw a provider with recent IYCF training than among clients who saw a provider without recent training (9% versus 3%), and counseling was more than twice as common among clients whose provider had any one type of training compared with clients whose provider had no training (7% versus 3%). In Malawi, counseling was higher for clients whose provider had received recent training in IYCF and whose provider had any training were counseled. However, the differences were not significant.

### Linked analysis: the relationship between the health service environment and early breastfeeding

We linked SPA facility-level data to DHS clusters for each service environment indicator. Additional file Table 2 presents, for Haiti and Malawi, the average number of facilities, average number of trained providers per facility, and average percentage of clients counseled in clusters among women with a recent live birth, by urban (or other urban) and rural place of residence.

Table 2 shows the sociodemographic background characteristics, as well as care-seeking and service environment characteristics by urban-rural residence and country. In Haiti, only 26% of rural women delivered in a health facility and by a skilled attendant; however, in both urban and rural women in Malawi, nearly all women received these services at delivery, 96 and 90%, respectively. In urban and rural settings of both countries, less than 40% of women reported PNC for either the mother or baby in the first hour. For the service environment characteristics, due to universally infrequent counseling, the lack of variation across clusters resulted in cluster classifications of “no data”, “low”, and “high” only. The category “no data” refers to areas with facilities offering ANC that either had no providers interviewed who provide ANC, or had no observations of women attending ANC on the day of the survey.

**Table 2:** Percent distribution of sociodemographic characteristics of women with a birth in the last 2 years

	Haiti^a^		Malawi	
	Other urban	Rural	Urban	Rural
**Region (Haiti)**				
Ouest	7.4	20.4		
Sud-Est	3.8	8.1		
Nord	22.1	10.6		
Nord-Est	10.3	3.9		
Artibonite	26.6	19.7		
Centre	10.0	10.4		
Sud	5.5	8.7		
Grand-Anse	6.3	6.2		
Nord-Ouest	6.5	7.8		
Nippes	1.5	4.3		
**Region (Malawi)**				
Northern			9.6	11.4
Central			40.6	42.7
Southern			49.8	45.9
**Wealth quintile**				
Lowest and second	11.6	68.9	4.8	55.6
Middle	28.5	21.0	6.0	21.9
Fourth and highest	60.0	10.2	89.1	22.5
**Education**				
None or primary	36.5	67.6	46.3	85.1
Secondary or higher	63.5	32.4	53.7	14.9
**Employment**				
Not employed	44.8	42.0	44.7	29.5
Employed	55.2	58.0	55.3	70.5
**Religion**				
Christian	89.7	85.9	34.0	29.5
Other	10.3	14.1	66.0	70.5
**Exposure to mass media**				
Less than once per week	66.6	69.1	43.1	69.9
At least once per week	33.4	30.9	56.9	30.1
**Currently married**				
No	17.4	13.0	16.5	16.2
Yes	82.6	87.0	83.5	83.8
**Parity**				
Primiparous	35.7	28.2	33.2	25.8
Multiparous	64.3	71.8	66.8	74.2
**Size of baby at birth**				
Normal	55.3	50.1	46.5	46.9
Small or very small	24.2	31.6	11.6	20.0
Large or very large	20.5	18.3	41.9	33.1
**Sex of child**				
Male	50.4	50.0	48.5	50.3
Female	49.6	50.0	51.5	49.7
**Number of ANC visits**				
One to three	16.2	36.0	44.2	52.9
Four or more	83.8	64.0	55.8	47.1
**Both SBA and facility delivery**				
Neither or just one	48.4	73.9	4.5	10.1
Both	51.6	26.1	95.5	89.9
**Mother or baby postnatal check in the first hour**				
No	64.7	79.5	61.1	62.3
Yes	35.3	20.5	38.9	37.7
**ANC facilities reporting breastfeeding counseling**				
No data	0.0	0.0	0.0	2.2
Low	40.4	35.7	31.9	54.6
Medium	33.1	33.0	32.0	19.8
High	26.5	31.3	36.0	23.3
**Access to trained providers**				
No data	0.0	0.0	0.0	2.8
Low	37.3	32.4	34.9	35.1
Medium	33.3	32.1	34.0	29.8
High	29.4	35.4	31.1	32.2
**Average facility breastfeeding counseling**				
No data	12.4	4.6	1.5	14.9
Low	63.6	67.6	66.9	67.7
High	24.0	27.8	31.7	17.4
**Number of clusters**	**92**	**284**	**171**	**675**
**Number of women**^**b**^	**357**	**1278**	**772**	**5296**

*ANC* antenatal care, *SBA* skilled birth attendant, *PNC* postnatal care

^a^Excludes urban women living in Port-au-Prince or rural women within 5 km of Port-au-Prince

^b^Includes women who had at least one ANC visit, who delivered vaginally, and whose baby survived past the day of birth

**Fig. 3:**
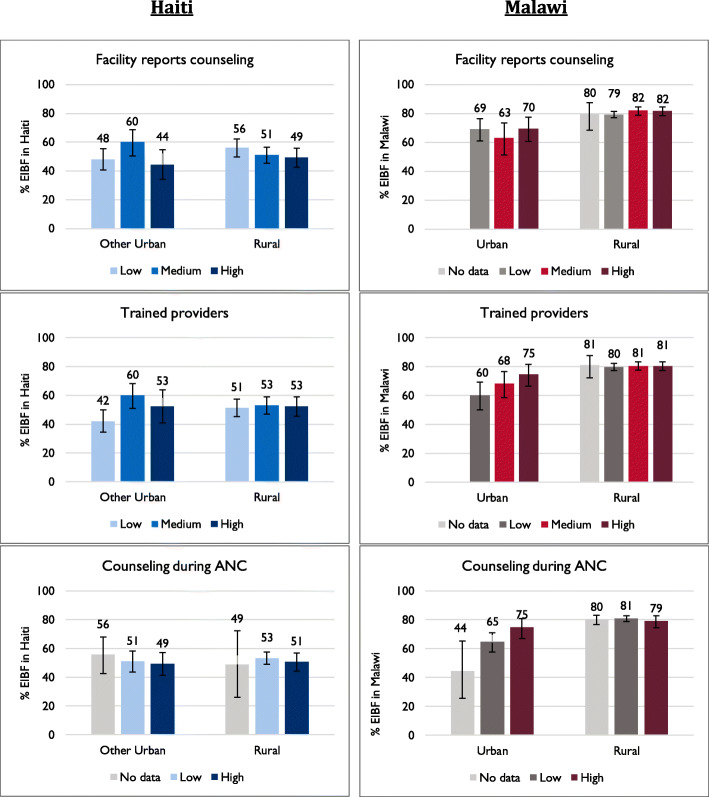
Breastfeeding in the first hour by health service environment factors, Haiti and Malawi

Figure 3 presents the proportion of women in Haiti and Malawi who practiced EIBF for their most recent birth, by each service environment factor. In Haiti, the only potential relationship appears between EIBF and having trained providers; in other urban areas, there is a difference of 18 percentage points in EIBF between women with low access to trained providers (42%) and women with medium access (60%), and a difference of 11 percentage points between having low access and high access (53%). Similarly, in urban areas of Malawi, 60% of women with low access to trained providers practiced EIBF compared with 68% of women with medium access and 75% of women with high access. Additionally, fewer urban women in clusters with no facilities offering ANC or with no ANC clients observed practiced EIBF (44%) than of women in clusters with a low level of counseling (65%) and high levels of counseling on breastfeeding (75%).

Table 3 shows the results of the adjusted multilevel logistic regression assessing the associations between early breastfeeding and the health service environment, as well as other sociodemographic and care-seeking covariates. Additional file Table 3 includes the unadjusted results. In Haiti, we found limited evidence of any relationships between service environment and EIBF. The only significant association identified between service environment and EIBF was for access to ANC providers with recent training on breastfeeding. In other urban areas of Haiti, women with medium and high access to trained providers had over twice the odds of EIBF compared with women with low access. This relationship was only statistically significant (*p*-value < 0.01) for women with medium access (AOR = 2.2, CI: 1.3–3.5) versus low access.

**Table 3:** Results of multivariable logistic regressions of early initiation of breastfeeding

	Haiti				Malawi			
	Other urban		Rural		Urban		Rural	
	AOR	95% CI	AOR	95% CI	AOR	95% CI	AOR	95% CI
**Department: Haiti (ref = Artibonite)**								
Ouest	0.7	0.2–2.8	0.8	0.5–1.5				
Sud-Est	0.6	0.2–2.4	0.6	0.3–1.1				
Nord	0.9	0.5–2.0	1.4	0.8–2.4				
Nord-Est	0.9	0.4–2.1	0.7	0.4–1.2				
Centre	0.8	0.4–1.9	0.4^**^	0.2–0.8				
Sud	0.3^*^	0.1–0.9	1.0	0.5–1.9				
Grand-Anse	2.7^*^	1.2–6.0	0.8	0.4–1.7				
Nord-Ouest	1.2	0.5–2.9	1.1	0.6–2.1				
Nippes	0.6	0.2–1.8	0.9	0.4–1.9				
**Region: Malawi (ref = Northern)**								
Central					0.2^***^	0.1–0.3	0.7^**^	0.5–0.9
Southern					0.4^*^	0.2–0.9	1.1	0.8–1.5
**Wealth quintile**								
Lowest and second	0.3^*^	0.1–0.8	ref		0.7	0.4–1.8	ref	
Middle	0.7	0.4–1.3	0.7	0.5–1.1	1.7	0.6–4.8	1.0	0.8–1.3
Fourth and highest	ref		0.5^*^	0.3–0.9	ref		0.9	0.7–1.2
**Education (ref = none or primary)**								
Secondary or higher	0.8	0.5–1.3	1.1	0.8–1.5	1.0	0.6–1.8	1.0	0.8–1.4
**Employment (ref = not employed)**								
Employed	1.1	0.7–2.0	1.4^**^	1.1–1.8	1.0	0.5–1.7	0.9	0.7–1.1
**Religion (ref = Christian)**								
Other	1.4	0.7–2.9	0.8	0.5–1.3	1.3	0.9–2.0	0.9	0.8–1.1
**Exposure to mass media (ref = less than once per week)**								
At least once per week	1.1	0.6–1.8	1.0	0.7–1.4	2.0^*^	1.1–3.7	1.0	0.8–1.2
**Currently married (ref = yes)**								
No	1.4	0.7–2.8	0.7	0.5–1.1	0.7	0.4–1.3	1.2	1.0–1.6
**Parity (ref = multiparous)**								
Primiparous	0.7	0.4–1.2	0.8	0.6–1.1	0.6	0.3–1.0	0.8^*^	0.6–1.0
**Size of baby at birth (ref = normal)**								
Small or very small	0.7	0.4–1.2	1.0	0.8–1.4	0.9	0.4–2.0	0.8	0.6–1.1
Large or very large	0.7	0.4–1.4	1.3	0.9–2.0	0.5^*^	0.3–0.9	0.9	0.7–1.0
**Sex of child (ref = female)**								
Male	0.9	0.5–1.4	1.0	0.8–1.2	1.7^*^	1.0–2.9	1.0	0.9–1.3
**Number of ANC visits (ref = one to three)**								
Four or more	0.5^*^	0.3–0.9	0.9	0.7–1.2	0.9	0.5–1.6	1.1	0.9–1.4
**Both SBA and facility delivery (ref = yes)**								
Neither or one or the other	0.8	0.4–1.7	1.0	0.6–1.5	0.3^*^	0.1–0.9	0.7^**^	0.5–0.9
**PNC in the first hour (ref = no)**								
Yes	1.9^*^	1.0–3.7	1.4	0.9–2.3	0.5^**^	0.3–0.8	1.2	1.0–1.5
**Access to ANC breastfeeding counseling (ref = low)**								
No data	n/a		n/a		n/a		0.8	0.3–1.7
Medium	1.3	0.8–2.2	0.7	0.4–1.0	0.7	0.4–1.2	1.1	0.8–1.4
High	0.8	0.4–2.0	0.6	0.4–1.0	0.9	0.5–1.6	1.0	0.7–1.3
**Access to trained providers (ref = low)**								
No data	n/a		n/a		n/a		1.3	0.7–2.4
Medium	2.2^**^	1.3–3.5	1.1	0.8–1.5	1.0	0.5–1.8	1.0	0.8–1.3
High	2.1	1.0–4.5	1.4	0.9–2.0	2.1^*^	1.1–4.0	1.0	0.8–1.3
**Average facility breastfeeding counseling in ANC (ref = low)**								
No data	1.7	0.9–3.3	0.6	0.2–2.0	0.3^**^	0.2–0.7	0.9	0.7–1.2
High	0.7	0.4–1.3	0.9	0.7–1.3	1.9^*^	1.0–3.5	0.9	0.7–1.2

*p<0.05, **p<0.01, ***p<0.001, *AOR* adjusted odds ratio, *CI* confidence interval, *n/a* not applicable, *ANC* antenatal care, *SBA* skilledbirth attendant, *PNC* postnatal care

In Malawi, we also identified a significant, positive relationship between EIBF and access to trained providers in urban areas. Women in urban clusters with high access to trained providers had over twice the odds of breastfeeding within the first hour compared with women with low access (AOR = 2.1, CI: 1.1–4.0). Additionally, urban women in clusters where clients were most often counseled on breastfeeding during ANC had nearly twice the odds of EIBF compared with women in clusters with the lowest level of counseling (AOR = 1.9, CI: 1.0–3.5), and urban women in clusters with “no data” (no facilities or facilities with observation of women attending ANC) were significantly less likely than women in clusters with low counseling on breastfeeding during ANC (AOR = 0.3, CI: 0.2–0.7). For counseling it should be noted that the very low levels overall contributed to little variation within facilities and within zones, to the extent that, due to skewness, it was only possible to dichotomize rather than use terciles for analysis.

We found mixed significant relationships across the two countries and by urban and rural residence between EIBF and care-seeking and other demographics, as shown in Table 3. In other urban areas of Haiti, women with four or more ANC visits (compared with one to three visits) had a 50% reduction in the odds of EIBF (AOR = 0.5, CI: 0.3–0.9), although PNC for the mother and baby was positively associated with EIBF (AOR = 1.9, CI: 1.0–3.7). Also, in rural areas of Haiti we found a reduced likelihood of EIBF among women in the wealthiest (fourth and fifth) household wealth quintiles (AOR = 0.5, CI: 0.3–0.9) compared with the lowest and second-lowest quintiles. Employed women in rural Haiti had increased odds of EIBF (AOR = 1.4, CI: 1.1–1.8) compared with unemployed women.

In Malawi, we found different associations between EIBF and care seeking as well as other demographic characteristics. Women in urban and rural areas who did not deliver with assistance by a skilled birth attendant (SBA) at a health facility were less likely to practice EIBF compared with women delivering in a facility with an SBA (urban AOR = 0.3, CI: 0.1–0.9; rural AOR = 0.7, CI: 0.5–0.9). However, women or babies who had a postnatal check in the first hour compared with those who did not were less likely to initiate breastfeeding within the first hour (AOR = 0.5, CI: 0.3–0.8). In urban Malawi, women who were exposed to the media at least once a week had a twofold increase in the odds of EIBF compared with women with media exposure less than once a week (AOR = 2.0, CI: 1.1–3.7). Women with large or very large babies at birth were less likely to practice EIBF compared with women with normal-sized babies (AOR = 0.5, CI: 0.3–0.9). In rural areas, women giving birth to their first child were less likely to breastfeed immediately compared with those who had also given birth previously (AOR = 0.8, CI: 0.6–1.0).

## Discussion

This study highlights the relationship between EIBF and the breastfeeding-related service environment in Haiti and Malawi. The study also reveals gaps in health facility readiness that can be addressed to improve breastfeeding counseling. Our results showed that although almost all facilities with ANC in Haiti and Malawi reported that they provided breastfeeding counseling as part of routine ANC services, less than 10% of clients received counseling on breastfeeding, regardless of trimester. Breastfeeding counseling is recommended for all pregnant women during ANC, but other studies have reported selective counseling during ANC due to low levels of provider counseling knowledge and skills as well as heavy workloads [7, 51–56].

Breastfeeding counseling in Haiti and Malawi is likely hindered by the shortage of skilled health providers [24, 25]. Where health personnel are already overburdened, providers often have little time to spend with clients [54, 56], thus counseling on breastfeeding or other topics that are part of recommended ANC practices is often neglected [52, 57]. Further, other studies examining quality of care during ANC in Rwanda and Mozambique have reported that health workers prioritize counseling on some topics, such as HIV, over others [52, 55, 57, 58]. Many countries have adopted task-shifting to reduce work overload, and have incorporated facility group counseling for breastfeeding and other maternal and child health interventions [59, 60]. Unfortunately, group counseling sessions were not captured in our study, thus we can only report on counseling received by the client from the provider during the ANC visit.

Provider training on breastfeeding counseling was positively associated with EIBF in both Haiti and Malawi. Providers who have undergone training for ANC spend more time on counseling [52]. Additionally, training can lead to improvements in counseling and, consequently, improved practices [52]. Antenatal counseling has been identified as an effective means to improve early breastfeeding outcomes [61, 62], yet training across a number of reproductive and maternal health topics including breastfeeding is not common [46, 63, 64]. Our study adds support to this as we observed a positive relationship between EIBF and training on counseling, yet less than half of providers in Haiti and Malawi had attended training on breastfeeding or IYCF in the preceding 24 months.

Our results showed that women’s report of receipt of maternal health services did not consistently predict EIBF across both countries in both urban and rural settings, such as ANC, SBA, and PNC. Several studies from different settings have shown that having skilled health workers is associated with higher prevalence of EIBF [11, 12, 65, 66]. In our study, however, these self-reported measures of healthcare contact (crude coverage of services) do not reflect the content or the circumstances of the care received. These factors are possibly influenced by confounding of unmeasured factors, such as pregnancy or birth complications that may have influenced care seeking, contact with the health system, and adoption of early breastfeeding. Further, effective coverage measures—that is, coverage adjusted for the quality of care—are proving to be robust determinants of nutrition-related outcomes (e.g., immediate breastfeeding) [67].

### Strengths and implications

This study took information from SPA health facility data on breastfeeding, which has been descriptively studied in recent research [63], and linked this information to data from the DHS surveys, providing a novel approach to study the association between ANC, the health service environment, and breastfeeding within an hour of birth. This methodology adds a health-systems perspective to research on breastfeeding that few studies have done before [11, 20], while taking into account differences between urban and rural areas in how the health service environment can relate to breastfeeding. Overall our results underscore the importance of the health facility service environment for breastfeeding outcomes, and add to extant research that the relationships between the two vary by urban and rural residence [8, 45].

### Limitations

A limitation of the study is the use of the aggregate measures of the service environment. In this facility-to-population linkage method, we cannot examine the direct relationship between a woman’s actual care received and her health outcomes, but rather the average care available at facilities and received among women in her area. These aggregate measures may be problematic when using provider and client variables, which we had already summarized within the facilities and then again within each zone around clusters.

Although our multilevel, multivariable analysis accounted for the nesting of individuals within clusters, we were not able to control for all individual, community, subnational, or national factors that might influence early breastfeeding. For example, while we controlled for birth weight, where low birth weight could serve as a proxy for preterm birth, we were unable to control for other complications that could impede early breastfeeding. We excluded neonatal deaths on the first day of life; however, near-miss cases and cases of maternal or newborn complications are unaccounted for in our analysis. We could not control for breastfeeding support and counseling from community health workers or cultural norms around breastfeeding within the community with any direct measure, nor did we incorporate higher-level factors such as large-scale or national programs or policies. Because of the cross-sectional nature of the data used in this study, we are unable to draw any causal inferences from our findings. Our analysis is limited to only Haiti and Malawi and may not be generalizable beyond these countries.

### Recommendations

Altogether, the findings of our analysis point to clear recommendations for ongoing and future policies and programs related to provider training. First, given the identified missed opportunities for the health system to ensure adoption of recommended breastfeeding practices (e.g., counseling about breastfeeding during ANC), more counseling is needed to meet WHO recommended ANC practices. Our findings suggest that provider training in breastfeeding counseling, which results in more counseling, can promote EIBF and likely other breastfeeding outcomes. Our study further points to a need for additional trainings for health workers. Additionally, emphasizing counseling during provider training is important, and training curricula should include specific guidance on breastfeeding counseling.

Based on our results, dedicated resources for scale-up of the Baby Friendly Hospital Initiative are also warranted in both Haiti and Malawi, especially to increase the availability of providers trained in breastfeeding counseling, although this recommendation should not be made without noting the additional burden that more time spent on counseling could mean for already overworked health providers. As a complement to facility heath workers, peer breastfeeding counseling through women’s groups and community-based health workers should continue to be used to improve EIBF; similarly, lay workers should receive appropriate training in breastfeeding counseling [7, 16, 68, 69].

Finally, to understand the role of the health system in promoting breastfeeding more globally, health facility surveys such as the SPA could be implemented in other countries. These surveys provide valuable information about the health system, including the quality of healthcare services, and when linked with household data, can help to elucidate the relationships between the health system and health outcomes.

## Conclusions

Initiation of breastfeeding within the first hour of birth has prodigious benefits for newborn health and survival, and counseling on breastfeeding during ANC can promote this behavior. While observed counseling during ANC was rare, the observation of ANC visits does not capture breastfeeding counseling that may occur in group education settings within the facility but apart from the ANC visit. Nonetheless, in urban Malawi, client receipt of breastfeeding counseling at nearby facilities was associated with early breastfeeding. However, we hypothesize that with more precise data that capture the counseling a woman directly receives, both during ANC and in the immediate postpartum period, a stronger and more ubiquitous relationship between counseling and early breastfeeding practices would be evidenced. Additionally, our study indicates a link between provider training and early breastfeeding among women in urban areas of Haiti and Malawi. These findings highlight and reinforce the importance of the Ten Steps to Successful Breastfeeding and the role of provider training on breastfeeding counseling.

## Supplementary information

**Additional file 1: Table S1.** Characteristics of health facilities that provide antenatal care services. Table depicting background characteristics of facilities. **Table S2.** Urban and rural cluster ranges of facility breastfeeding readiness and counseling**.** Table depicting the urban and rural service environments. **Table S3.** Results of unadjusted logistic regressions of early initiation of breastfeeding. Table depicting bivariate regression results.

## Data Availability

All data including Demographic and Health Surveys and Service Provision Assessment data are publicly available from https://dhsprogram.com/data/available-datasets.cfm.

## Abbreviations

ANC: Antenatal care
AOR: Adjusted odds ratio
CI: Confidence interval
DHS: Demographic and Health Surveys
EIBF: Early Initiation of Breastfeeding
PNC: Postnatal care
PTSD: Posttraumatic stress disorder
SBA: Skilled birth attendant
UOR: Unadjusted odds ratio
WHO: World Health Organization

## References

[1] Rollins NC, Bhandari N, Hajeebhoy N, Horton S, Lutter CK, Martines JC, Piwoz EG, Richter LM, Victora CG (2016). Why invest, and what it will take to improve breastfeeding practices?. Lancet.

[2] Victora CG, Bahl R, Barros AJ, Franca GV, Horton S, Krasevec J, Murch S, Sankar MJ, Walker N, Rollins NC (2016). Breastfeeding in the 21st century: epidemiology, mechanisms, and lifelong effect. Lancet.

[3] WHO (2017). Guideline: protecting, promoting and supporting breastfeeding in facilities providing maternity and newborn services.

[4] Neovita Study Group (2016). Timing of initiation, patterns of breastfeeding, and infant survival: prospective analysis of pooled data from three randomised trials. Lancet Glob Health.

[5] Smith ER, Hurt L, Chowdhury R, Sinha B, Fawzi W, Edmond KM, Neovita Study G (2017). Delayed breastfeeding initiation and infant survival: a systematic review and meta-analysis. PLoS One.

[6] UNICEF Data: Monitoring the situation of children and women: Infant and young child feeding. https://data.unicef.org/topic/nutrition/infant-and-young-child-feeding/. Accessed 1 July 2019.

[7] WHO (2018). Guideline: counselling of women to improve breastfeeding practices.

[8] Adewuyi EO, Zhao Y, Khanal V, Auta A, Bulndi LB (2017). Rural-urban differences on the rates and factors associated with early initiation of breastfeeding in Nigeria: further analysis of the Nigeria demographic and health survey, 2013. Int Breastfeed J.

[9] Benedict RK, Craig HC, Torlesse H, Stoltzfus RJ (2018). Trends and predictors of optimal breastfeeding among children 0–23 months, South Asia: analysis of national survey data. Matern Child Nutr.

[10] Berde AS, Yalcin SS (2016). Determinants of early initiation of breastfeeding in Nigeria: a population-based study using the 2013 demograhic and health survey data. BMC Pregnancy Childbirth.

[11] Khan SM, Speizer IS, Singh K, Angeles G, Twum-Danso NA, Barker P (2017). Does postnatal care have a role in improving newborn feeding? A study in 15 sub-Saharan African countries. J Glob Health.

[12] Ogbo FA, Eastwood J, Page A, Efe-Aluta O, Anago-Amanze C, Kadiri EA, Ifegwu IK, Woolfenden S, Agho KE (2017). The impact of sociodemographic and health-service factors on breast-feeding in sub-Saharan African countries with high diarrhoea mortality. Public Health Nutr.

[13] Patel A, Bucher S, Pusdekar Y, Esamai F, Krebs NF, Goudar SS, Chomba E, Garces A, Pasha O, Saleem S (2015). Rates and determinants of early initiation of breastfeeding and exclusive breast feeding at 42 days postnatal in six low and middle-income countries: a prospective cohort study. Reprod Health.

[14] Prior E, Santhakumaran S, Gale C, Philipps LH, Modi N, Hyde MJ (2012). Breastfeeding after cesarean delivery: a systematic review and meta-analysis of world literature. Am J Clin Nutr.

[15] Bhattacharjee NV, Schaeffer LE, Marczak LB, Ross JM, Swartz SJ, Albright J, Gardner WM, Shields C, Sligar A, Schipp MF (2019). Mapping exclusive breastfeeding in Africa between 2000 and 2017. Nat Med.

[16] Sinha B, Chowdhury R, Sankar MJ, Martines J, Taneja S, Mazumder S, Rollins N, Bahl R, Bhandari N (2015). Interventions to improve breastfeeding outcomes: a systematic review and meta-analysis. Acta Paediatr.

[17] Beake S, Pellowe C, Dykes F, Schmied V, Bick D (2012). A systematic review of structured compared with non-structured breastfeeding programmes to support the initiation and duration of exclusive and any breastfeeding in acute and primary health care settings. Matern Child Nutr.

[18] Perez-Escamilla R, Martinez JL, Segura-Perez S (2016). Impact of the baby-friendly hospital initiative on breastfeeding and child health outcomes: a systematic review. Matern Child Nutr.

[19] Gupta A, Suri S, Dadhich JP, Trejos M, Nalubanga B (2019). The world breastfeeding trends initiative: implementation of the global strategy for infant and young child feeding in 84 countries. J Public Health Policy.

[20] Takahashi K, Ganchimeg T, Ota E, Vogel JP, Souza JP, Laopaiboon M, Castro CP, Jayaratne K, Ortiz-Panozo E, Lumbiganon P (2017). Prevalence of early initiation of breastfeeding and determinants of delayed initiation of breastfeeding: secondary analysis of the WHO global survey. Sci Rep.

[21] Donabedian A (1988). The quality of care: how can it be assessed?. JAMA.

[22] Donabedian A (1966). Evaluating the quality of medical care. Milbank Mem Fund Q.

[23] Burgert CR, Prosnitz D (2014). Linking DHS household and SPA facility surveys: data considerations and geospatial methods. DHS spatial analysis reports no 10.

[24] United Nations Development Programme and Oxford Poverty and Human Development Initiative (2019). Global multidimensional poverty index 2019: illuminating inequalities.

[25] WHO (2019). Global spending on health: a world in transition (No. WHO/HIS/HGF/HFWorkingPaper/19.4).

[26] Haiti Health Workforce Data Audits Lead to Human Resources for Health Improvements**.** https://www.hfgproject.org/health-workforce-data-audits-lead-improvements-human-resources-health-haiti/#_edn1. Accessed 1 July 2019.

[27] IHE, ICF International (2014). Haïti Évaluation de la Prestation des Services de Soins de Santé 2013.

[28] WHO (2017). National implementation of the Baby-friendly Hospital Initiative.

[29] PAHO (2016). The baby friendly hospital initiative in Latin America and the Caribbean: current status, challenges, and opportunities.

[30] DHS STATCompiler. http://statcompiler.com/en/. Accessed 1 July 2019.

[31] Republic of Haiti (2013). Plan Stratégique National de Nutrition 2013–2018.

[32] Scaling Up Nutrition (2017). Haiti: institutional transformations 2016–2017.

[33] MoH Malawi, ICF International (2014). Malawi service provision assessment 2013–14.

[34] Kavle JA, Welch PR, Bwanali F, Nyambo K, Guta J, Mapongo N, Straubinger S, Kambale S (2019). The revitalization and scale-up of the Baby-Friendly Hospital Initiative in Malawi. Matern Child Nutr.

[35] Government of Malawi (2018). Malawi national multi-sector nutrition policy 2018–2022.

[36] Government of Malawi (2009). National Nutrition Policy and Strategic Plan.

[37] Malawi Ministry of Health Nutrition Unit (2008). The world breastfeeding trends initiative: Malawi assessment report.

[38] Cayemittes M, Busangu MF, Bizimana J, Barrere B, Severe B, Cayemittes V, Charles E (2013). Enquête Mortalité, Morbidité et Utilisation des Services EMMUS-V: Haiti.

[39] NSO/Malawi, ICF (2017). Malawi demographic and health survey 2015–16.

[40] Rowe-Murray HJ, Fisher JR (2002). Baby friendly hospital practices: cesarean section is a persistent barrier to early initiation of breastfeeding. Birth.

[41] Do M, Micah A, Brondi L, Campbell H, Marchant T, Eisele T, Munos M (2016). Linking household and facility data for better coverage measures in reproductive, maternal, newborn, and child health care: systematic review. J Glob Health.

[42] Assaf S, Wang W, Mallick L (2016). Provider counseling and knowledge transfer in health facilities of Haiti, Malawi, and Senegal. DHS analytical studies no 60.

[43] Bessinger RE, Bertrand JT (2001). Monitoring quality of care in family planning programs: a comparison of observations and client exit interviews. Int Fam Plan Perspect.

[44] Hameed W, Ishaque M, Gul X, Siddiqui J-u-R, Hussain S, Hussain W, Ahmed A, Balal A (2017). Does courtesy bias affect how clients report on objective and subjective measures of family planning service quality? A comparison between facility-and home-based interviews. Open Access J Contracept.

[45] Wang W, Winter R, Mallick L, Florey L, Burgert-Brucker C, Carter E (2015). The relationship between the health service environment and service utilization: linking population data to health facilities data in Haiti and Malawi. DHS analytical studies no 51.

[46] Mallick L, Temsah G, Wang W (2019). Comparing summary measures of quality of care for family planning in Haiti, Malawi, and Tanzania. PLoS One.

[47] Leslie HH, Sun Z, Kruk ME (2017). Association between infrastructure and observed quality of care in 4 healthcare services: a cross-sectional study of 4,300 facilities in 8 countries. PLoS Med.

[48] IHSI (2015). Population totale, population de 18 ans et plus ménages et densités estimés en 2015.

[49] Gage AD, Leslie HH, Bitton A, Jerome JG, Thermidor R, Joseph JP, Kruk ME (2017). Assessing the quality of primary care in Haiti. Bull World Health Organ.

[50] WHO. WHO antenatal care randomized trial: manual for the implementation of the new model. Geneva: World Health Organization; 2002.

[51] WHO (2016). WHO recommendations on antenatal care for a positive pregnancy experience.

[52] Magoma M, Requejo J, Merialdi M, Campbell OM, Cousens S, Filippi V (2011). How much time is available for antenatal care consultations? Assessment of the quality of care in rural Tanzania. BMC Pregnancy Childbirth.

[53] Ngabo F, Zoungrana J, Faye O, Rawlins B, Rosen H, Levine R, Sethi R, MacDowell J, Arscott-Mills S, Basinga P (2012). Quality of care for prevention and management of common maternal and newborn complications: findings from a national health facility survey in Rwanda.

[54] Phillips E, Stoltzfus RJ, Michaud L, Pierre GLF, Vermeylen F, Pelletier D (2017). Do mobile clinics provide high-quality antenatal care? A comparison of care delivery, knowledge outcomes and perception of quality of care between fixed and mobile clinics in central Haiti. BMC Pregnancy Childbirth.

[55] Rurangirwa AA, Mogren I, Ntaganira J, Govender K, Krantz G (2018). Quality of antenatal care services in Rwanda: assessing practices of health care providers. BMC Health Serv Res.

[56] Solnes Miltenburg A, van der Eem L, Nyanza EC, van Pelt S, Ndaki P, Basinda N, Sundby J (2017). Antenatal care and opportunities for quality improvement of service provision in resource limited settings: a mixed methods study. PLoS One.

[57] von Both C, Fleβa S, Makuwani A, Mpembeni R, Jahn A (2006). How much time do health services spend on antenatal care? Implications for the introduction of the focused antenatal care model in Tanzania. BMC Pregnancy Childbirth.

[58] Biza A, Jille-Traas I, Colomar M, Belizan M, Harris JR, Crahay B, Merialdi M, Nguyen MH, Althabe F, Aleman A (2015). Challenges and opportunities for implementing evidence-based antenatal care in Mozambique: a qualitative study. BMC Pregnancy Childbirth.

[59] Conrad P, Schmid G, Tientrebeogo J, Moses A, Kirenga S, Neuhann F, Müller O, Sarker M (2012). Compliance with focused antenatal care services: do health workers in rural Burkina Faso, Uganda and Tanzania perform all ANC procedures?. Tropical Med Int Health.

[60] Lewin S, Munabi-Babigumira S, Glenton C, Daniels K, Bosch-Capblanch X, van Wyk BE, Odgaard-Jensen J, Johansen M, Aja GN, Zwarenstein M. Lay health workers in primary and community health care for maternal and child health and the management of infectious diseases. Cochrane Database Syst Rev. 2010;(3)1–170. 10.1002/14651858.CD004015.pub3 PMC6485809 20238326

[61] Imdad A, Yakoob MY, Bhutta ZA (2011). Effect of breastfeeding promotion interventions on breastfeeding rates, with special focus on developing countries. BMC Public Health.

[62] Wouk K, Tully KP, Labbok MH (2017). Systematic review of evidence for baby-friendly hospital initiative step 3: prenatal breastfeeding education. J Hum Lact.

[63] Mallick L, Temsah G, Benedict RK (2018). Facility-based nutrition readiness and delivery of maternal and child nutrition services using service provision assessment surveys. DHS comparative reports no 49.

[64] Wang W, Mallick L, Allen C, Pullum T (2019). Effective coverage of facility delivery in Bangladesh, Haiti, Malawi, Nepal, Senegal, and Tanzania. PLoS One.

[65] Bergamaschi N, Oakley L, Benova L (2019). Is childbirth location associated with higher rates of favourable early breastfeeding practices in sub-Saharan Africa?. J Glob Health.

[66] Sharma S, Van Teijlingen E, Hundley V, Angell C, Simkhada P (2016). Dirty and 40 days in the wilderness: eliciting childbirth and postnatal cultural practices and beliefs in Nepal. BMC Pregnancy Childbirth.

[67] Joseph NT, Piwoz E, Lee D, Malata A, Leslie HH (2020). Examining coverage, content, and impact of maternal nutrition interventions: the case for quality-adjusted coverage measurement. J Glob Health.

[68] Chapman DJ, Morel K, Anderson AK, Damio G, Perez-Escamilla R (2010). Breastfeeding peer counseling: from efficacy through scale-up. J Hum Lact.

[69] Perry H, Zulliger R (2012). How effective are community health workers. An overview of current evidence with recommendations for strengthening community health worker programs to accelerate progress in achieving the health-related millennium development goals.

