# Supplementary material for: Facility readiness and counseling during antenatal care and the relationship with early breastfeeding in Haiti and Malawi
Source: BMC Pregnancy Childbirth. 2020 May 29;20:325. doi: 10.1186/s12884-020-02919-7 (PMC7257126; doi:10.1186/s12884-020-02919-7)
Supplement: Supplementary file 1 — Additional file 1: Table S1. Characteristics of health facilities that provide antenatal care services. Table depicting background characteristics of facilities. Table S2. Urban and rural cluster ranges of facility breastfeeding readiness and counseling. Table depicting the urban and rural service environments. Table S3. Results of unadjusted logistic regressions of early initiation of breastfeeding. Table depicting bivariate regression results. [file 12884_2020_2919_MOESM1_ESM.docx]

Additional file

Table 1. Characteristics of health facilities that provide antenatal care services

|  | Haiti | | Malawi | |
| --- | --- | --- | --- | --- |
|  | Urban | Rural | Urban | Rural |
| **Type of facility^1^** |  |  |  |  |
| Hospital | 25.8 | 4.2 | 44.4 | 9.9 |
| Health center | 64.5 | 37.2 | 24.4 | 80.8 |
| Dispensary, clinic, other | 9.7 | 58.6 | 31.3 | 9.2 |
| **Managing authority** |  |  |  |  |
| Government | 33.0 | 43.7 | 48.3 | 66.5 |
| Private not-for-profit | 37.1 | 38.5 | 22.1 | 25.9 |
| Private for profit, other | 30.0 | 17.8 | 29.6 | 7.6 |
| **Region (Haiti)** |  |  |  |  |
| Aire Métropolitain / Reste-Ouest | 54.8 | 24.3 |  |  |
| Sud-Est | 4.2 | 9.6 |  |  |
| Nord | 9.3 | 8.8 |  |  |
| Nord-Est | 1.6 | 5.5 |  |  |
| Artibonite | 4.5 | 18.0 |  |  |
| Centre | 3.5 | 6.3 |  |  |
| Sud | 9.7 | 6.1 |  |  |
| Grand-Anse | 4.8 | 4.8 |  |  |
| Nord-Ouest | 4.0 | 13.3 |  |  |
| Nippes | 3.5 | 3.4 |  |  |
| **Region (Malawi)** |  |  |  |  |
| North |  |  | 16.0 | 19.2 |
| Central |  |  | 40.8 | 36.3 |
| South |  |  | 43.2 | 44.5 |
| **Total** | **310** | **522** | **116** | **516** |
| **^1^** In Haiti, hospitals are university, regional, community, and other hospitals; and health centers include those with and without beds. In Malawi, hospitals are central, district, community, and other hospitals; health centers include maternity health centers; and other refers to health posts. | | | | |

**Table 2. Urban and rural cluster ranges of facility breastfeeding readiness and counseling**

|  | Haiti^1^ | | | | | | Malawi | | | | | |
| --- | --- | --- | --- | --- | --- | --- | --- | --- | --- | --- | --- | --- |
|  | Other Urban | | | Rural | | | Urban | | | Rural | | |
|  | Low | Medium | High | Low | Medium | High | Low | Medium | High | Low | Medium | High |
| Number of ANC facilities with reporting routine counseling on breastfeeding^2^ | 1 - 2 | 3 - 5 | 6 - 22 | 1 - 5 | 6 - 9 | 10 - 23 | 0 - 3 | 4 - 7 | 8 - 17 | 0 - 2 | 3 | 4 - 18 |
| Average number of providers trained on breastfeeding^2, 3^ | 0 - 0.4 | 0.4 - 0.6 | 0.6 - 2.6 | 0 - 0.3 | 0.3 - 0.5 | 0.5 - 2.9 | 0 - 0.2 | 0.2 - 0.5 | 0.5 - 4.3 | 0 - 0.3 | 0.3 - 0.6 | 0.6 - 3.4 |
| Percent of clients counseled on breastfeeding^4^ | 0% | n/a | 1% - 60% | 0% | n/a | 1% - 100% | 0% | n/a | 1% - 33% | 0% | n/a | 3% - 100% |
|  | | | | | | | | | | | | |
| ^1^ Excludes urban clusters in Port-au-Prince or rural clusters with 5 km of Port-au-Prince  ^2^ Includes only clusters with women who had at least one ANC visit, who delivered vaginally, and whose baby survived past the day of birth  ^3^ Cut-off points appear overlap due to rounding  ^4^Includes only clusters with at least one linked facility with antenatal care client data in addition to inclusion criteria above | | | | | | | | | | | | |
|  | | | | | | | | | | | | |

Table 3. Unadjusted logistic regressions of early breastfeeding for the last birth in the last 2 years

|  | Haiti | | | | Malawi | | | |
| --- | --- | --- | --- | --- | --- | --- | --- | --- |
|  | Other urban | | Rural | | Urban | | Rural | |
| Variables | UOR | 95% CI | UOR | 95% CI | UOR | 95% CI | UOR | 95% CI |
| **Department: Haiti (ref=Artibonite)** |  |  |  |  |  |  |  |  |
| Ouest | 0.8 | 0.2 - 3.3 | 0.8 | 0.4 - 1.7 |  |  |  |  |
| Sud-Est | 1.4 | 0.4 - 5.0 | 1.5 | 0.8 - 2.7 |  |  |  |  |
| Nord | 1.5 | 0.4 - 6.1 | 1.0 | 0.5 - 2.1 |  |  |  |  |
| Nord-Est | 2.3 | 0.6 - 8.2 | 1.2 | 0.7 - 2.2 |  |  |  |  |
| Centre | 1.6 | 0.4 - 6.2 | 0.9 | 0.5 - 1.6 |  |  |  |  |
| Sud | 0.6 | 0.2 - 2.4 | 1.2 | 0.7 - 2.1 |  |  |  |  |
| Grand-Anse | 2.4 | 0.6 - 8.8 | 1.3 | 0.6 - 2.5 |  |  |  |  |
| Nord-Ouest | 1.2 | 0.3 - 5.1 | 1.3 | 0.7 - 2.4 |  |  |  |  |
| Nippes | 1.6 | 0.3 - 8.3 | 1.2 | 0.6 - 2.5 |  |  |  |  |
| **Region: Malawi (ref=Northern)** |  |  |  |  |  |  |  |  |
| Central |  |  |  |  | 0.3*** | 0.1 - 0.5 | 0.6*** | 0.5 - 0.8 |
| Southern |  |  |  |  | 0.4** | 0.2 - 0.8 | 1.1 | 0.8 - 1.4 |
| **Wealth quintile** |  |  |  |  |  |  |  |  |
| Lowest and second | **0.5*** | 0.2 - 1.0 | ref |  | 0.5 | 0.2 - 1.3 | ref |  |
| Middle | 0.9 | 0.5 - 1.6 | 0.8 | 0.6 - 1.1 | 1.0 | 0.4 - 2.6 | 1.0 | 0.8 - 1.3 |
| Fourth and highest | ref |  | 0.6* | 0.3 - 0.9 | ref |  | 1.0 | 0.8 - 1.3 |
| **Education (ref=none or primary)** |  |  |  |  |  |  |  |  |
| Secondary or higher | 0.9 | 0.6 - 1.3 | 0.9 | 0.7 - 1.2 | 1.1 | 0.6 - 1.8 | 1.0 | 0.8 - 1.3 |
| **Employment (ref=not employed)** |  |  |  |  |  |  |  |  |
| Employed | 1.3 | 0.8 - 2.1 | 1.5** | 1.2 - 1.9 | 0.9 | 0.5 - 1.5 | 0.9 | 0.7 - 1.1 |
| **Religion (ref=Christian)** |  |  |  |  |  |  |  |  |
| Other | 1.5 | 0.7 - 3.0 | 0.8 | 0.5 - 1.2 | 1.3 | 0.8 - 2.1 | 1.0 | 0.9 - 1.2 |
| **Exposure to mass media  (ref=less than once per week)** |  |  |  |  |  |  |  |  |
| At least once per week | 1.0 | 0.6 - 1.7 | 1.0 | 0.7 - 1.4 | 2.0** | 1.2 - 3.4 | 1.0 | 0.8 - 1.2 |
| **Currently Married (ref=yes)** |  |  |  |  |  |  |  |  |
| No | 1.1 | 0.5 - 2.3 | 0.7* | 0.5 - 1.0 | 0.7 | 0.4 - 1.3 | 1.2 | 0.9 - 1.5 |
| **Parity (ref=multiparous)** |  |  |  |  |  |  |  |  |
| Primiparous | 0.7 | 0.5 - 1.1 | 0.8 | 0.6 - 1.0 | 0.6 | 0.4 - 1.0 | 0.8 | 0.7 - 1.0 |
| **Size of baby at birth (ref=normal)** |  |  |  |  |  |  |  |  |
| Small or very small | 0.7 | 0.4 - 1.1 | 1.0 | 0.8 - 1.4 | 0.7 | 0.3 - 1.7 | 0.8 | 0.6 - 1.0 |
| Large or very large | 0.8 | 0.4 - 1.4 | 1.3 | 0.9 - 1.9 | 0.6* | 0.3 - 0.9 | 0.9 | 0.7 - 1.1 |
| **Sex of child (ref=female)** |  |  |  |  |  |  |  |  |
| Male | 0.9 | 0.6 - 1.5 | 0.9 | 0.8 - 1.2 | 1.5 | 0.9 - 2.6 | 1.0 | 0.9 - 1.3 |
| **Number of ANC visits (ref=one to three)** | |  |  |  |  |  |  |  |
| Four or more | 0.7 | 0.4 - 1.3 | 0.9 | 0.7 - 1.1 | 0.9 | 0.6 - 1.5 | 1.2 | 0.9 - 1.4 |
| **Both SBA and facility delivery  (ref=yes)** |  |  |  |  |  |  |  |  |
| Neither or one or the other | 0.7 | 0.4 - 1.1 | 1.0 | 0.7 - 1.3 | 0.4 | 0.1 - 1.4 | 0.6** | 0.5 - 0.9 |
| **PNC in the first hour (ref=no)** |  |  |  |  |  |  |  |  |
| Yes | **1.8**** | 1.2 - 2.7 | 1.3 | 0.9 - 1.9 | 0.6* | 0.3 - 0.9 | 1.2* | 1.0 - 1.5 |
|  |  |  |  |  |  |  |  |  |
| **Service environment variables** |  |  |  |  |  |  |  |  |
| **Access to breastfeeding counseling  in ANC (ref=low)** |  |  |  |  |  |  |  |  |
| No data | n/a |  | n/a |  | n/a |  | 1.0 | 0.5 - 2.1 |
| Medium | 1.6 | 1.0 - 2.6 | 0.8 | 0.6 - 1.1 | 0.8 | 0.4 - 1.6 | 1.2 | 0.9 - 1.5 |
| High | 0.9 | 0.5 - 1.4 | 0.8 | 0.5 - 1.1 | 1.0 | 0.5 - 1.9 | 1.1 | 0.9 - 1.5 |
| **Access to trained providers (ref=low)** |  |  |  |  |  |  |  |  |
| No data | n/a |  | n/a |  | n/a |  | 1.1 | 0.6 - 2.0 |
| Medium | 2.1** | 1.3 - 3.3 | 1.1 | 0.7 - 1.5 | 1.5 | 0.8 - 2.9 | 1.0 | 0.8 - 1.3 |
| High | 1.5 | 0.8 - 2.7 | 1.1 | 0.7 - 1.5 | 2.1* | 1.1 - 4.0 | 1.1 | 0.8 - 1.4 |
| **Average facility breastfeeding counseling in ANC (ref=low)** |  |  |  |  |  |  |  |  |
| No data | 1.2 | 0.6 - 2.3 | 0.8 | 0.3 - 2.6 | 0.4* | 0.2 - 0.9 | 0.9 | 0.7 - 1.2 |
| High | 0.9 | 0.6 - 1.5 | 0.9 | 0.7 - 1.3 | 1.6 | 1.0 - 2.8 | 0.9 | 0.6 - 1.2 |
|  | | | | | | | | |
| *p<0.05; **p<0.01; ***p<0.001; UOR= unadjusted odds ratio; CI=confidence interval; ANC = antenatal Care; SBA = skilled birth attendance; PNC = postnatal care | | | | | | | | |
|  | | | | | | | | |
